# Paratesticular desmoplastic small round cell tumour: an unusual tumour with an unusual fusion; cytogenetic and molecular genetic analysis combining RT-PCR and COBRA-FISH

**DOI:** 10.1186/2045-3329-2-3

**Published:** 2012-01-25

**Authors:** Vincent PM Cliteur, Károly Szuhai, Hans J Baelde, Jurriaan van Dam, Hans Gelderblom, Pancras CW Hogendoorn

**Affiliations:** 1 Department of Pathology, Westfries Gasthuis, Hoorn NH, The Netherlands; 2 Department of Molecular Cell Biology, Leiden University Medical Center, Einthovenweg 20, Leiden, The Netherlands; 3 Department of Pathology, Leiden University Medical Center, Leiden, Albinusdreef 2, Leiden, The Netherlands; 4 Department of Clinical Oncology, Leiden University Medical Center, Albinusdreef 2, Leiden, The Netherlands

**Keywords:** soft tissue tumour, desmoplastic small round cell tumour, paratesticular, treatment, molecular pathology

## Abstract

Desmoplastic small round cell tumour is a rare malignant tumour with a male to female ratio of 4:1. It manifests mostly at serosal sites. Here we present a case of a 28-year-old male patient, who presented with a fast growing paratesticular mass. On biopsy nests and cords of small round cells, without a clear morphological lineage of differentiation were seen. Occasionally desmoplatic small round cell tumour shows different lines of differentiation. An unequivocal histological diagnosis might be difficult in such cases. Here we demonstrate by a combination of methods the characteristic immunohistochemical profile and - albeit unusual - molecular background and discuss the eventual link with Ewing sarcoma.

Immunohistochemical studies showed a membranous staining of Keratine AE1/3 and a dot-like staining of Desmine, confirming its diagnosis. Using COBRA-FISH following a metaphase approach we demonstrated a balanced translocation, t(11;22)(p13;q12) and in RT-PCR formation of the EWSR1-WT1 fusion product, a specific translocation of desmoplastic round cell tumour. The fusion involves exon 9 of EWSR1 to exon 8 of WT1, an unusual fusion product, though earlier described in a case of a desmoplastic small round cell tumour of the hand. The EWSR1-WT1 chimera seems to function as an oncogenic transcription factor. Here the zinc finger domain of the WT1 acts with affinity with certain promoter domains influencing the expression of various downstream proteins such as: PDGFA, PAX2, insulin-like growth factor 1 receptor, epidermal growth factor receptor, IL2 receptor beta, BAIAP3, MLF1, TALLA-1, LRRC15 and ENT. We discuss their potential oncogenic roles and potential therapeutic consequences.

## Introduction

Desmoplastic small round cell tumour (DSRT) was first described as a distinct entity by Gerald and Rosai now about 23 years ago [1]. The tumour is nowadays well-characterized histologically and at the immunohistochemical level and involves serosal surfaces. Microscopically, the morphologic prototype of the tumour shows nests of small, round cells embedded within a desmoplastic stroma, giving it its descriptive name as an entity. The cells show a multidirectional phenotype with epithelial, myogenic and neural marker expression. The tumour is located in the abdominal and pelvic peritoneal cavity in the vast majority of cases. Extra-abdominal sites are rare. Cases were described however in the cranial cavity, lung, head and neck, salivary gland [2], limbs, pancreas and paratesticular region. Although most cases have been described in young men and children, several cases have been described in older patients [3-5] and in women, sometimes simulating ovarian tumours [3,4,6], even with high CA125 levels [6,7].

DSRCT is associated with a distinct translocation which shows a fusion between Ewing sarcoma gene (EWSR1) and Wilms tumour gene (WT1). There are at least two reports of hybrid tumours with features of both DSRCT and Ewing sarcoma, one with an EWSR1-FLI1 fusion gene [8] and one with an EWSR1-ERG fusion gene [6]. Rapid growth and metastasis to liver, lungs, pleura, bone, spleen and lymph nodes [9,3] is common. The reported prognosis is poor, with a median survival of 17 months only [9-15]. At the meeting of the Connective Tissue Oncology Society, November 2010, Subbiah et al presented data from 161 patients, with a median survival duration of 2.4 years.

Long-term survivors are uncommon and therapeutical possibilities are limited, although reports of debulking combined with chemotherapy with high-dose multi-agent alkylator-based systemic therapy mention response rates and improved progression-free survival. A study of Schwartz and co-workers [16] showed a progression-free survival of 18% at five years, indicating a very poor prognosis. Factors correlated with improved survival include a complete or very good partial response to therapy with the so-called P6 protocol; and a greater than 90% tumour resection. The commonly used Memorial Sloan-Kettering P6 protocol consists of four courses of high-dose cyclophosphamide, doxorubicin, and vincristine, interspersed with ifosfamide, etoposide, and mesna in three cycles. In addition, some patients in the literature underwent myeloablative chemotherapy with stem cell rescue and/or radiation therapy. DSRCTs are generally sensitive to chemotherapy and radiotherapy, however their response is rarely lasting. Therefore, surgical resection is considered to be of primary importance for achieving prolonged disease-free survival [16].

Lal et al [14] studied 66 patients and found gross tumour resection significant in prolonging the overall survival. In the case of resectable tumours the 3-year survival was 58%, while in the case of non-resectable tumour localizations the 3-year survival was 0%.

Livaditi et al. [15] found in a combination of radical tumour excision with adjuvant chemotherapy, all had recurrence within 2-6 months. Gil et al [12] observed no correlation between the surgical excision and improved survival rates.

In this report, we present a rare case of desmoplastic round cell tumour presenting as a paratesticular mass and with cytogenetic analysis using a multicolour Combined Binary RAtio labelling Fluorescence In-situ Hybridization (COBRA-FISH) method and with a rare translocation variant detected by reverse transcriptase-polymerase chain reaction (RT-PCR) analysis of the t(11;22) fusion gene product.

## Materials and methods

### Case report

A 32-year-old male presented in a referral hospital with a distinct paratesticular scrotal mass, without other complaints. On biopsy a diagnosis of desmoplastic small round cell tumour was suggested. A subsequently performed CT-scan showed extensive localization in the abdomen.

Orchidectomy was performed. Subsequently, following the diagnosis of DSRCT, the patient was treated with chemotherapy: three cycles VIDE: Vincristine 1 mg/m^2^, Etoposide 150 mg/m^2^, Doxorubicine 20 mg/m^2^, Ifosfamide 3000 mg/m^2^. After three cycles chemotherapy, an incomplete debulking procedure was performed and post-operatively another three cycles VIDE were administered. A complete resection was not possible, because of tumour extension in the abdomen. The patient returned back to work and was relatively well for one and a half year. Unfortunately the patient died 24 months later with progressive metastases in the liver and abdominal cavity.

### Immunohistochemistry

Immunohistochemistry was performed on the biopsy as well as on the debulking specimen using antibodies directed against desmin (Dako, Glostrup, Denmark) and CD99 (Dako), CD56 (Dako) and EMA (Dako) staining, according to standard laboratory protocols.

### Tumour sample and chromosome preparation

A fresh sample of tumour tissue was minced and subsequently inoculated into a culture flask containing 5 ml of RPMI 1640 medium 1 mg/ml collagenase 1A (sigma-Aldrich) and 1 mg/ml dispase (GIBCO) to enzymatically disaggregate cells. After 16 hrs. of treatment, released tumour cells were washed and cultured in RPMI 1640 medium, supplemented with 10% fetal calf serum and penicillin/streptomycin. After five days of incubation, metaphase cells were harvested using colcemid (20 ng/ml) and incubated for two hrs. After trypsinization and hypotonic treatment (0.075 M KCl for 12 min) cells were fixed by three changed fixation steps using methanol/glacial acetic acid (3:1 v/v). The cell suspension was dropped and selected slides were used for COBRA-FISH analysis.

### Molecular Cytogenetic Analysis

Slides with metaphase chromosomes were hybridized using a multicolour FISH approach named COBRA-FISH. A 48-colour FISH staining every chromosome-arm in a different colour combination, digital imaging and analysis were performed as previously described by our group [17,18]. Hybridization with individual libraries labelled with single fluorochromes was used to confirm the detected rearrangements. Breakpoints were assigned using inverted 4,6-diamidino-2-phenylindole (DAPI) counterstained images of the chromosomes. Karyotypes have been described according to the International System for Human Cytogenetic Nomenclature (ISCN), 2009.

### RT-PCR

RNA isolation, reverse transcription, PCR and gel electrophoresis were performed as described earlier. RNA was extracted from fresh frozen tumour tissue with TRIzol (Gibco BRL Life Technologies, Gaithersburg, MD, USA). RNA was reversely transcribed in a mix containing 2 μg RNA and 0.2 μl AMV-reverse transcriptase (Boehringer Mannheim, Germany). Amplification by PCR was carried out using 1 U Ampli-Taq polymerase (Perkin-Elmer Thermal cycler. A total of 30 cycles were performed of 30 sec at 94°C (denaturation), 60 sec at 68°C (annealing) and 60 sec at 72°C (elongation). An assay that uses a forward EWSR1 primer (5' EWSR1: EWS-ex7 for 5'-TGTAAAACGACGGCCAGTtcctacagccaagctccaagtc-3') and a reverse WT1 primer (3' WT1: WT1-ex9rev 5'-CAGGAAACAGCTATGACCgaccaggagaactttcgctgac-3'), was performed to detect EWSR1/WT1 chimerical transcripts.

## Results

### Histopathology

**Figure 1:**
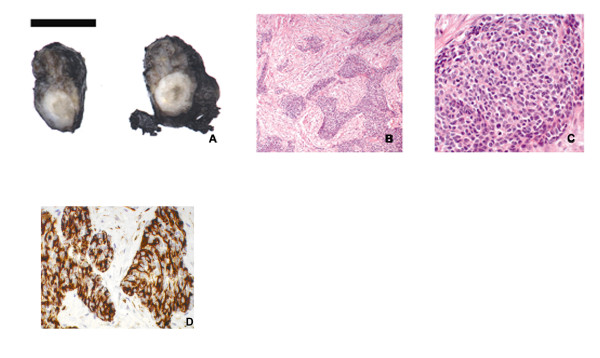
**Macro- and microscopic features**. A: gross specimen. The tumour masses were solid, firm, with grey-white cut surfaces and areas of necrosis. B: light micrograph showing compact nests and sheets of small, round cells in desmoplastic stroma. Magnification 25× C: numerous mitotic figures can be observed. Magnification 200× D: Immunohistochemical detection of desmin; the reaction shows a typical paranuclear dot-like staining. Magnification 200×.

Sections obtained from the testicular tumour mass (Figure 1A) were composed of sharply defined nests, sheets and cords of undifferentiated cells (Figure 1B and 1C) tumour cells have scant cytoplasm, indistinct cell borders and round to oval nuclei. Mitotic figures were numerous, up to five per high power field. Apoptosis and necrosis were prominent. Extensive desmoplastic stroma was present.

### Immunohistochemistry

Desmin immunohistochemistry showed a positive staining in a paranuclear dot-like fashion (Figure 1D), while CD56 and EMA showed a diffuse membranous staining and CD99 a faint membranous staining.

### Molecular Cytogenetic Analysis

**Figure 2:**
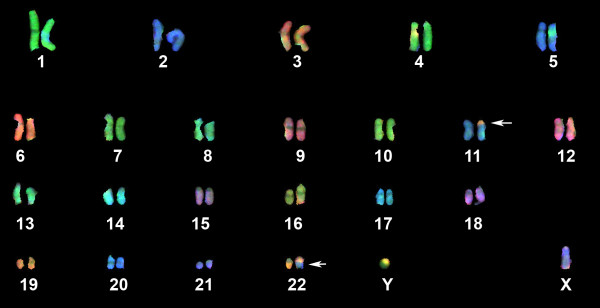
**COBRA-FISH karyogram of the case showing the characteristic t(11;22)(p13;q12) as a solely alteration**. Rearranged chromosomes are indicated by arrowheads.

Multicolour FISH-based karyotyping is capable of showing each individual arm of every chromosome. A representative karyotype image is shown in Figure 2. The following karyotype was ascertained: 46, XY, t(11;22)(p13;q12), no secondary chromosomal alteration were detected.

### RT-PCR

Detection of the EWSR1-WT1 fusion transcript by RT-PCR showed a fusion product in the expected range. The sequence analysis showed a fusion product of exon 9 EWSR1 with exon 8 of WT1.

## Discussion

Desmoplastic small round cell tumour (DSCRT) is a rare malignant tumour, sometimes with a distinct histological appearance and less frequently with different growth patterns. A pure morphological diagnosis is sometimes difficult and molecular techniques can be helpful to differentiate between other poorly differentiated tumours. The typical presentation is with a paraserosal mass and a predilection for serosal surfaces, mainly the peritoneum and - as in our case - the paratesticular region. DSCRT shows a histological pattern with small cells growing in a nested pattern with abundant, desmoplastic stroma [4,5]. Several atypical patterns have been described with rhabdoid, clear cell, pleomorphic, glandular, basaloid patterns, solid areas with little stroma, pseudorosettes, fusiform and papillary areas [4,19-21].

The immunohistochemical profile is characterized by the co-expression of epithelial, mesenchymal and neural markers (table 1). Gerald et al. [4] suggested that DSRCT is a primitive tumour related to mesothelium, because of the prevalence in serosal cavities. The tumour growth pattern mimics that of mesothelioma. Fetal mesothelium co-expresses Keratin and Desmin. Frequent WT1 expression has been reported in mesothelioma. Calretinin, a protein highly expressed in normal mesothelium as well as in mesothelioma, is curiously negative however in DSRCT [22].

**Table 1:** Antibody profiles in the literature

Antibody	Gerald, Ordonez and Lae (*)		**Zhang et al**. [22]	
Desmin	135/149	90.6%	21/23	91%

Vimentin	86/93	92.5%	NT	

NSE	105/131	80.2%	NT	

WT1	62/68	91.2%	16/23	70%

Keratin (not specified)	132/149	88.6%	NT	

CAM5.2	NT		21/23	91%

kerAE1AE3	NT		16/23	70%

EMA	74/79	93.7%	NT	

CD99	NT		13/23	57%

Muscle specific actin	2/89	2.2%	NT	

S-100	6/30	20%	NT	

Synaptophysin	3/19	15.8%	NT	

Chromogranin	2/68	2.9%	NT	

PLAP	NT		17/21	81%

Myoglobine	NT		5/17	29%

Myogenine	0/18	0%	0/22	0%

Calretinine	NT		4/21	19%

HER2	NT		7/18	39%

c-kit	NT		2/14	14%

(*) literature data are retrieved from Gerald et al [36], Ordonez [21] and Lae et al. [25]. Desmin and vimentin show a typical paranuclear dotlike staining. The target genes (PDGF-A, PDGFR beta en TGF-beta3) are almost all positive. NT: Not Tested

At the genomic level, it is not entirely clear whether the EWSR1-WT1 translocation is a specific translocation for DSRCT or not. Alaggio et al. described two pediatric cases with morphological, and immunohistochemical features of leiomyosarcomas with a EWSR1-WT1 translocation. Surprisingly, the behaviour of these tumours seemed to have a favourable course. The presence of this translocation in leiomyosarcomas suggests that the fusion of EWSR1-WT1 is not entirely specific for DSRCT [23]. Wang et al. described 4 children with renal tumours with EWSR1-WT1 fusion and typical histological and immunohistochemical features of DSRCT, but without the desmoplastic stroma. Curiously, the patients in this study have a much lower age of presentation than the average and their prognosis seems to be better than usually described for DSRCT's [24].

At genomic level, DSRCT is associated with a recurrent chromosomal translocation, t(11;22)(p13;q12) forming an in-frame chimera transcript. At the protein level a fusion of the amino-terminal domain (NTD) of the EWSR1 gene and three of the four carboxyl-terminal zinc fingers of the WT1 tumour suppressor gene takes place [19]. A specific chromosomal translocation, t(11;22)(p12;q12), has been identified in DSRCT in 93% of all cases [25]. The diagnostic dilemma in those cases is between the phenotype and the genotype. There are at least two reports of hybrid tumours with features of both DSRCT and Ewing sarcoma, one with a EWSR1-FLI1 fusion gene [8] and one with a EWSR1-ERG fusion gene [6]. Their classification is therefore enigmatic. As in Ewing sarcoma, the chimerical protein included the EWSR1 N-terminal domain and the SP3 zinc finger DNA-binding domain but not the inhibitory domain of SP3-domain of WT1. The first zinc finger protein involved, also acts as a transcriptional activator of repressor. There are a number of similarities between both tumours: the age of presentation is similar and histologically both tumours show small, undifferentiated round cells. The immunophenotype is however different. In the case of ES/PNET usually a restricted or unidirectional differentiation toward neural elements is seen, whereas DSRCT shows a divergent differentiation. There is a large overlap: both Keratin and occasionally Desmin can be expressed in ES/PNET. Conversely, CD99 expression is also found in DSRCT, as in our case. This phenomenon emphasizes the importance of molecular research to distinquish the different tumors, especially because of the more distinguish therapies [26].

The classical fusion in DSCRT is EWSR1 exon 7/WT1 exon 8. Translocation variants of EWSR1-WT1, in decreasing reported frequencies are: three cases with a EWSR1 exon 9-WT1 exon 8, two cases with a EWSR1 exon 10-WT1 exon 8, one case with a EWSR1 exon 7-WT exon 8 [27], one case with a EWSR1 exon 5 -WT1 exon 10 [24] and one case with EWSR1 exon 9-WT1 exon 8 [28]. In all cases the junctions produced in-frame transcripts. The case presented here shows a rare fusion between EWSR1 exon 9- WT1 exon 8, similar to that reported by Adsay et al of a DSCRT of the hand. The translocation breakpoint within the EWSR1 gene occurs in intron 9 retaining of the amino-terminal domain (NTD) of EWSR1 that fuses zinc fingers 2-4 to the NTD of WT1. WT1 is a zinc-finger protein, with specific DNA- and RNA-binding domains. The translocation event in DSRCT abolishes the RNA-binding activity of EWSR1, as well as the transcriptional repression activity of WT1. Werner et al. [29] showed that WT1 binds to specific cis-elements in the IGF-IR gene regulatory region and suppresses IGF-IR gene transcription.

The fusion proteins are believed to function as strong transcription factors, with direct targets PDGFA, Pax2, insulin-like growth factor 1 receptor (IGF-IR), epidermal growth factor receptor, IL2 receptor beta, BAIAP3, MLF1, TALLA-1, and LRRC15 [29-32].

IGF-IR is a potent anti-apoptotic receptor tyrosine kinase [25]. IL2 receptor beta is promoted by the isoform EWSR1-WT1 (-KTS), whereas the desmoplastic stroma expresses IL2 and IL15, so functions as a paracrine growth signal [31,32]. BAIAP3, this protein is believed to participate in regulated exocytosis and expressed in tumour cells of DSRCT and enhance tumour growth by secretion of autocrine or paracrine growth factors [33]. MLF1 is induced approximately eight-fold by EWSR1-WT1 (-KTS) and plays a role in proliferation and cell survival [30]. TALLA1 induction regulates interactions with extracellular matrix, migration, and invasion. LRRC15 by EWSR1-WT1 (+KTS) is strongly expressed in invasive breast cancer cell line and possibly contributes to the invasive phenotype of DSCRT [34]. Some of these genes might serve as target for therapy, especially when novel treatment modalities are investigated [35]. Different strategies for therapy are summarized in table 2, with chemotherapy, radiotherapy, stem cell transplantation and with antibodies for example against the insulin-like growth factor 1 receptor (AMG 479).

**Table 2:** Clinical trials

NCT ID	Drugs
00417807	Imatinib mesylate

01189643	Iriontecan, temozolomide, bevacizumab incorporated into an existing schedule of high dose alkylator.

00563680	AMG-479

00055952	Exatecan mesylate

01277744	Hyperthermic peritoneal perfusion with cisplatin.

01099644	Radioimmunotherapy.

00062205	Imatinib mesylate

00436657	Hyperthermic perfusion with cisplatin.

01287104	NK cell infusion following allogeneic stem cell transplantation.

01125449	Ascorbic acid (vitamin C)

00445965	Iodine I131 monoclonal antibody 3F8.

00720174	IMC-A12, doxorubicine hydrochloride.

00003926	Amifostine to protect form side effects of PSCT.

00002515	Chemotherapy in combination with bone marrow transplantation.

00024258	Arsenic trioxide.

00043979	Stem cell transplantation.

00562380	AMG-479.

01154452	GDC-0449 and RO4929097.

00025441	Combination chemotherapy.

00794521	Pazopanib.

00474994	Sunitinib malate

00526149	BI 2536

00002466	Combination chemotherapy and radiation therapy.

00030667	Imatinib mesylate.

00089245	Iodine I131 monoclonal antibody 8H9.

00093821	Tanespimycin

00662233	Combination chemotherapy

00002898	Surgery followed by chemotherapy.

01189253	Doxorubicin or Trabectedin.

In conclusion, here we report a detailed molecular characterization of DSCRT with a rare translocation variant forming a junction between the EWSR1 exon 9 and WT1 exon 8 at the transcript level. The functional consequences of this variant in terms of dysregulation at the target gene level, with potential implication of novel treatment modalities, need additional investigation.

## Consent

Samples were obtained according to the ethical guidelines of the host institution. Samples were analysed in a coded fashion and all procedures were performed according to the ethical guidelines "Code for Proper Secondary Use of Human Tissue in The Netherlands" (Dutch Federation of Medical Scientific Societies).

## Competing interests

The authors declare that they have no competing interests.

## Authors' contributions

**VPMC: **participated in conceiving the study, in microscopy, in literature study and drafted the manuscript.

**KS: **participated in the design of the study, carried out the molecular genetic studies and drafted the manuscript

**JVD: **participated in the design, microscopy and literature study and molecular genetic studies.

PCWH: participated in conceiving the study, diagnosed the case, corrected the manuscript and supervised the clinical molecular studies

JJB: drafted the RT-PCR testing and subsequently performed all PCR based assays; corrected the manuscript.

AJG: provided clinical details and follow up, arranged consent, participated in the literature study and corrected the manuscript

All authors have read and approved the final version of the manuscript file.

## References

[B1] Gerald WL Rosai J Case 2 Desmoplastic small cell tumor with divergent differentiation. Pediatr Pathol 1989 9 2 177 83 10.3109/15513818909022347 2473463

[B2] Yin WH Guo SP Yang HY Chan JK Desmoplastic small round cell tumor of the submandibular gland: a rare but distinctive primary salivary gland neoplasm Hum Pathol 2010 41 3 438 42 10.1016/j.humpath.2009.08.015 19913282

[B3] Cummings OW Ulbright TM Young RH Del Tos AP Fletcher CD Hull MT Desmoplastic small round cell tumors of the paratesticular region. A report of six cases Am J Surg Pathol 1997 21 2 219 25 10.1097/00000478-199702000-00013 9042290

[B4] Gerald WL Miller HK Battifora H Miettinen M Silva EG Rosai J Intra-abdominal desmoplastic small round-cell tumor. Report of 19 cases of a distinctive type of high-grade polyphenotypic malignancy affecting young individuals Am J Surg Pathol 1991 15 6 499 513 10.1097/00000478-199106000-00001 1709557

[B5] Ordonez NG el Naggar AK Ro JY Silva EG Mackay B Intra-abdominal desmoplastic small cell tumor: a light microscopic, immunocytochemical, ultrastructural, and flow cytometric study Hum Pathol 1993 24 8 850 65 10.1016/0046-8177(93)90135-4 8375856

[B6] Ordi J de Alava E Torne A Mellado B Pardo-Mindan J Iglesias X Intraabdominal desmoplastic small round cell tumor with EWS/ERG fusion transcript Am J Surg Pathol 1998 22 8 1026 32 10.1097/00000478-199808000-00014 9706984

[B7] Ordonez NG Sahin AA CA 125 production in desmoplastic small round cell tumor: report of a case with elevated serum levels and prominent signet ring morphology Hum Pathol 1998 29 3 294 9 10.1016/S0046-8177(98)90050-8 9496834

[B8] Thorner P Intra-abdominal polyphenotypic tumor Pediatr Pathol Lab Med 1996 16 1 161 9 10.1080/107710496176010 8963628

[B9] Saab R Khoury JD Krasin M Davidoff AM Navid F Desmoplastic small round cell tumor in childhood: the St. Jude Children's Research Hospital experience Pediatr Blood Cancer 2007 49 3 274 9 10.1002/pbc.20893 16685737

[B10] Amato RJ Ellerhorst JA Ayala AG Intraabdominal desmoplastic small cell tumor. Report and discussion of five cases Cancer 1996 78 4 845 51 10.1002/(SICI)1097-0142(19960815)78:4<845::AID-CNCR22>3.0.CO;2-U 8756380

[B11] Bertuzzi A Castagna L Quagliuolo V Ginanni V Compasso S Magagnoli M Prospective study of high-dose chemotherapy and autologous peripheral stem cell transplantation in adult patients with advanced desmoplastic small round-cell tumor Br J Cancer 2003 89 7 1159 61 10.1038/sj.bjc.6601304 14520438 PMC2394317

[B12] Gil A Gomez PA Brun EA Sugarbaker PH Clinical perspective on desmoplastic small round-cell tumor Oncology 2004 67 3-4 231 42 10.1159/000081323 15557784

[B13] Hassan I Shyyan R Donohue JH Edmonson JH Gunderson LL Moir CR Intraabdominal desmoplastic small round cell tumors: a diagnostic and therapeutic challenge Cancer 2005 104 6 1264 70 10.1002/cncr.21282 16080179

[B14] Lal DR Su WT Wolden SL Loh KC Modak S La Quaglia MP Results of multimodal treatment for desmoplastic small round cell tumors J Pediatr Surg 2005 40 1 251 5 10.1016/j.jpedsurg.2004.09.046 15868593

[B15] Livaditi E Mavridis G Soutis M Papandreou E Moschovi M Papadakis V Diffuse intraabdominal desmoplastic small round cell tumor: a ten-year experience Eur J Pediatr Surg 2006 16 6 423 7 10.1055/s-2006-924736 17211792

[B16] Schwarz RE Gerald WL Kushner BH Coit DG Brennan MF La Quaglia MP Desmoplastic small round cell tumors: prognostic indicators and results of surgical management Ann Surg Oncol 1998 5 5 416 22 10.1007/BF02303860 9718171

[B17] Tanke HJ Wiegant J van Gijlswijk RP Bezrookove V Pattenier H Heetebrij RJ New strategy for multi-colour fluorescence in situ hybridisation: COBRA: COmbined Binary RAtio labelling Eur J Hum Genet 1999 7 1 2 11 10.1038/sj.ejhg.5200265 10094185

[B18] Szuhai K Tanke HJ COBRA: combined binary ratio labeling of nucleic-acid probes for multi-color fluorescence in situ hybridization karyotyping Nat Protoc 2006 1 1 264 75 10.1038/nprot.2006.41 17406243

[B19] Gerald WL Rosai J Ladanyi M Characterization of the genomic breakpoint and chimeric transcripts in the EWS-WT1 gene fusion of desmoplastic small round cell tumor Proc Natl Acad Sci USA 1995 92 4 1028 32 10.1073/pnas.92.4.1028 7862627 PMC42630

[B20] Ordonez NG Desmoplastic small round cell tumor: II: an ultrastructural and immunohistochemical study with emphasis on new immunohistochemical markers Am J Surg Pathol 1998 22 11 1314 27 10.1097/00000478-199811000-00002 9808124

[B21] Ordonez NG Desmoplastic small round cell tumor: I: a histopathologic study of 39 cases with emphasis on unusual histological patterns Am J Surg Pathol 1998 22 11 1303 13 10.1097/00000478-199811000-00001 9808123

[B22] Zhang PJ Goldblum JR Pawel BR Fisher C Pasha TL Barr FG Immunophenotype of desmoplastic small round cell tumors as detected in cases with EWS-WT1 gene fusion product Mod Pathol 2003 16 3 229 35 10.1097/01.MP.0000056630.76035.F3 12640103

[B23] Alaggio R Rosolen A Sartori F Leszl A d'Amore ES Bisogno G Spindle cell tumor with EWS-WT1 transcript and a favorable clinical course: a variant of DSCT, a variant of leiomyosarcoma, or a new entity? Report of 2 pediatric cases Am J Surg Pathol 2007 31 3 454 9 10.1097/01.pas.0000213375.02171.43 17325488

[B24] Wang LL Perlman EJ Vujanic GM Zuppan C Brundler MA Cheung CR Desmoplastic small round cell tumor of the kidney in childhood Am J Surg Pathol 2007 31 4 576 84 10.1097/01.pas.0000213432.14740.14 17414105

[B25] Lae ME Roche PC Jin L Lloyd RV Nascimento AG Desmoplastic small round cell tumor: a clinicopathologic, immunohistochemical, and molecular study of 32 tumors Am J Surg Pathol 2002 26 7 823 35 10.1097/00000478-200207000-00001 12131150

[B26] Romeo S Dei Tos AP Hogendoorn PCW The clinical impact of molecular techniques on diagnostic pathology of soft tissue and bone tumours Diagnostic Histopathology 2012 18 2 81 85 10.1016/j.mpdhp.2011.11.004

[B27] Yamaguchi U Hasegawa T Morimoto Y Tateishi U Endo M Nakatani F A practical approach to the clinical diagnosis of Ewing's sarcoma/primitive neuroectodermal tumour and other small round cell tumours sharing EWS rearrangement using new fluorescence in situ hybridisation probes for EWSR1 on formalin fixed, paraffin wax embedded tissue J Clin Pathol 2005 58 10 1051 6 10.1136/jcp.2004.025502 16189150 PMC1770737

[B28] Adsay V Cheng J Athanasian E Gerald W Rosai J Primary desmoplastic small cell tumor of soft tissues and bone of the hand Am J Surg Pathol 1999 23 11 1408 13 10.1097/00000478-199911000-00012 10555010

[B29] Werner H Idelman G Rubinstein M Pattee P Nagalla SR Roberts CT Jr A novel EWS-WT1 gene fusion product in desmoplastic small round cell tumor is a potent transactivator of the insulin-like growth factor-I receptor (IGF-IR) gene Cancer Lett 2007 247 1 84 90 10.1016/j.canlet.2006.03.027 16730884

[B30] Gerald WL Haber DA The EWS-WT1 gene fusion in desmoplastic small round cell tumor Semin Cancer Biol 2005 15 3 197 205 10.1016/j.semcancer.2005.01.005 15826834

[B31] Wong JC Lee SB Bell MD Reynolds PA Fiore E Stamenkovic I Induction of the interleukin-2/15 receptor beta-chain by the EWS-WT1 translocation product Oncogene 2002 21 13 2009 19 10.1038/sj.onc.1205262 11960373

[B32] Zhang PJ Goldblum JR Pawel BR Pasha TL Fisher C Barr FG PDGF-A, PDGF-Rbeta, TGFbeta3 and bone morphogenic protein-4 in desmoplastic small round cell tumors with EWS-WT1 gene fusion product and their role in stromal desmoplasia: an immunohistochemical study Mod Pathol 2005 18 3 382 7 10.1038/modpathol.3800264 15389255

[B33] Palmer RE Lee SB Wong JC Reynolds PA Zhang H Truong V Induction of BAIAP3 by the EWS-WT1 chimeric fusion implicates regulated exocytosis in tumorigenesis Cancer Cell 2002 2 6 497 505 10.1016/S1535-6108(02)00205-2 12498718

[B34] Reynolds PA Smolen GA Palmer RE Sgroi D Yajnik V Gerald WL Identification of a DNA-binding site and transcriptional target for the EWS-WT1(+KTS) oncoprotein Genes Dev 2003 17 17 2094 107 10.1101/gad.1110703 12923058 PMC196452

[B35] Oka Y Tsuboi A Oji Y Kawase I Sugiyama H WT1 peptide vaccine for the treatment of cancer Curr Opin Immunol 2008 20 2 211 20 10.1016/j.coi.2008.04.009 18502632

[B36] Gerald WL Ladanyi M de Alava E Cuatrecasas M Kushner BH Laquaglia MP Clinical, pathologic, and molecular spectrum of tumors associated with t(11;22)(p13;q12): desmoplastic small round-cell tumor and its variants J Clin Oncol 1998 16 9 3028 36 9738572 10.1200/JCO.1998.16.9.3028

